# Development of a multi-gene expression system in *Xanthophyllomyces dendrorhous*

**DOI:** 10.1186/s12934-014-0175-3

**Published:** 2014-12-04

**Authors:** Kiyotaka Y Hara, Toshihiko Morita, Masao Mochizuki, Keisuke Yamamoto, Chiaki Ogino, Michihiro Araki, Akihiko Kondo

**Affiliations:** Organization of Advanced Science and Technology, Kobe University, 1-1 Rokkodaicho Nada-ku, Kobe, 657-8501 Japan; Department of Chemical Science and Engineering, Graduate School of Engineering, Kobe University, 1-1 Rokkodaicho Nada-ku, Kobe, 657-8501 Japan

**Keywords:** Isoprenoid, Carotenoid, Astaxanthin, Mevalonate, *Xanthophyllomyces dendrorhous*, *Phaffia rhodozyma*, Metabolic engineering, Cell factory

## Abstract

**Background:**

Red yeast, *Xanthophyllomyces dendrorhous* (*Phaffia rhodozyma*) is the only yeast known to produce astaxanthin, an anti-oxidant isoprenoid (carotenoid) that is widely used in the aquaculture, food, pharmaceutical and cosmetic industries. Recently, the potential of this microorganism as a platform cell factory for isoprenoid production has been recognized because of high flux through its native terpene pathway. Addition of mevalonate, the common precursor for isoprenoid biosynthesis, has been shown to be critical to enhance the astaxanthin content in *X. dendrorhous*. However, addition of mevalonate is unrealistic during industrial isoprenoid production because it is an unstable and costly chemical. Therefore, up-regulating the intracellular mevalonate supply by enhancing the mevalonate synthetic pathway though genetic engineering is a promising strategy to improve isoprenoid production in *X. dendrorhous*. However, a system to strongly express multiple genes has been poorly developed for *X. dendrorhous*.

**Results:**

Here, we developed a multiple gene expression system using plasmids containing three strong promoters in *X. dendrorhous* (actin, alcohol dehydrogenase and triose-phosphate isomerase) and their terminators. Using this system, three mevalonate synthetic pathway genes encoding acetoacetyl-CoA thiolase, HMG-CoA synthase and HMG-CoA reductase were overexpressed at the same time. This triple overexpressing strain showed an increase in astaxanthin production compared with each single overexpressing strain. Additionally, this triple overexpression of mevalonate synthetic pathway genes together with genes involved in β-carotene and astaxanthin synthesis showed a synergetic effect on increasing astaxanthin production. Finally, astaxanthin production was enhanced by 2.1-fold compared with the parental strain without a reduction of cell growth.

**Conclusions:**

We developed a system to strongly overexpress multiple genes in *X. dendrorhous*. Using this system, the synthetic pathway of mevalonate, a common substrate for isoprenoid biosynthesis, was enhanced, causing an increase in astaxanthin production. Combining this multiple gene overexpression system with a platform strain that overproduces mevalonate has the potential to improve industrial production of various isoprenoids in *X. dendrorhous*.

**Electronic supplementary material:**

The online version of this article (doi:10.1186/s12934-014-0175-3) contains supplementary material, which is available to authorized users.

## Background

Carotenoids are widely distributed in nature, and are exclusively synthesized by plants and microorganisms [1]. Carotenoids belong to the natural compounds class of terpenes (isoprenoids) [2]. Bioproduction of pharmaceutically important carotenoids such as artemisinin and Taxol have been accomplished through genetic engineering of well-characterized microorganisms such as *Saccharomyces cerevisiae* and *Escherichia coli* [3,4]. However, Melillo, *et al*., showed the potential of the red yeast, *Phaffia rhodozyma* (sexual form, *Xanthophyllomyces dendrorhous*), as a platform microorganism for isoprenoids production because of the higher flux through its native terpene pathway compared with *S. cerevisiae* and *E. coli* [5].

**Figure 1:**
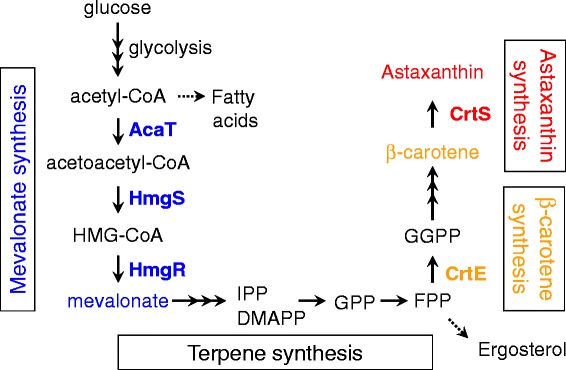
**Metabolic pathway from glucose to astaxanthin in** ***X. dendrorhous*** **.** The pathway consists of the glycolytic pathway and mevalonate, terpene, β-carotene and astaxanthin synthetic pathways. IPP, isopentenyl-pyrophosphate; DMAPP, dimethylallyl diphosphate; GPP, geranyl diphosphate; FPP, farnesyl-diphosphate; GGPP, geranylgeranyl-diphosphate; AcaT, acetoacetyl-CoA thiolase; HmgS, HMG-CoA synthase; HmgR, HMG-CoA reductase; CrtE, geranylgeranyl-diphosphate synthase; CrtS, astaxanthin synthase. IPP and DMAPP are the isoprenoid building blocks and prenyltransferases generate GPP, FPP and GGPP, the common precursors of monoterpenoids (C10), sesquiterpenoids (C15) and diterpenoids (C20), respectively. Carotenoids (C40) such as astaxanthin are constructed by condensation of two molecules of GGPP.

*X. dendrorhous* has been studied as a promising candidate microorganism for maximizing production of a carotenoid, astaxanthin (3, 3′-dihydroxy-β, β-carotene-4, 4′-dione; C_40_H_52_O_4_) for use for fine chemicals such as food, pharmaceuticals and cosmetics because of its antioxidant property [6,7]. In fungi, including *X. dendrorhous*, all isoprenoids are biosynthesized from a common precursor, mevalonate, via the terpene pathway (Figure 1). It has been shown that supplementation of mevalonate was critical to increase the astaxanthin content in *X. dendrorhous* [8]. However, mevalonate addition is not economically feasible for industrial production because it is an unstable and expensive chemical. Thus, increasing the supply of intracellular mevalonate by metabolic engineering would be a valuable way to increase isoprenoid production because it negates the need and the cost of externally added mevalonate. It has already been reported that reducing the carbon flow to the fatty acid and ergosterol biosynthesis pathways (Figure 1) enhances astaxanthin production due to the increase in carbon flow to mevalonate biosynthesis pathway [9]. However, this made cell growth slower because fatty acids and ergosterol are important structural components of the cell membrane. This suggests that strengthening the mevalonate synthetic pathway by overexpressing genes encoding enzymes involved in the mevalonate synthetic pathway is a promising approach to enhance biosynthesis of astaxanthin or any other isoprenoids without additional compounds and effects on cell growth in *X. dendrorhous*. Indeed, it has already been reported that overexpressing *hmgR*, which is one of genes involved in the mevalonate synthetic pathway, under its original promoter was critical to enhance the astaxanthin content in *X. dendrorhous* [10].

We previously identified several strong promoters of *X. dendrorhous* using GFP as a protein expression level indicator [7]. In this study, we developed a system to overexpress multiple genes under these strong promoters in *X. dendrorhous*. Overexpression of three genes involved in the mevalonate synthetic pathway using this system improved astaxanthin production in *X. dendrorhous*. Furthermore, simultaneous overexpression of these mevalonate synthetic pathway genes with genes involved in the β-carotene and astaxanthin synthetic pathways synergistically enhanced the biosynthesis of astaxanthin; this production of astaxanthin indicates the ability of this strain to produce high quantities of isoprenoids. Combining the mevalonate producing strain and the multiple gene overexpression system developed in this study gives us powerful tools to improve production of various isoprenoids in *X. dendrorhous*.

## Results and discussion

### Single gene overexpression of mevalonate synthetic pathway genes

**Figure 2:**
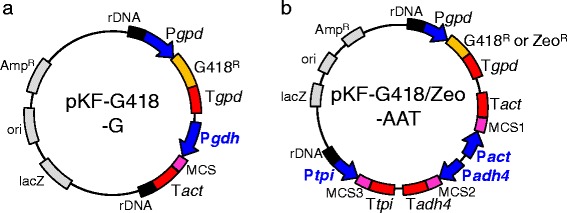
**Vectors for overexpression of single or multiple target genes. a** vector for overexpression of a single gene. **b** vector for overexpression of multiple genes. rDNA, ribosomal DNA sequence; MCS, multi-cloning site; G418^R^, G418 resistance gene; Zeo^R^, Zeocin resistance gene; P*gpd* and T*gpd*, glycerol-3-phosphate dehydrogenase promoter and terminator; P*gdh*, glutamate dehydrogenase promoter; P*act* and T*act*, actin promoter and terminator; P*adh4* and T*adh4*, alcohol dehydrogenase 4 promoter and terminator; P*tpi* and T*tpi*, triose-phosphate isomerase promoter and terminator.

We constructed single gene overexpression vectors to enhance the mevalonate synthetic pathway (Figure 2a). As shown in Figure 1, mevalonate biosynthesis from acetyl-CoA is carried out by three consecutive reactions catalyzed by acetoacetyl-coenzyme A (acetoacetyl-CoA) thiolase (AcaT, EC 2.3.1.194), 3-hydroxy-3-methylglutaryl-CoA (HMG-CoA) synthase (HmgS, EC 2.3.3.10), and HMG-CoA reductase (HmgR, EC 1.1.1.34). AcaT catalyzes the acetoacetyl-CoA biosynthetic reaction from acetyl-CoA, HmgS catalyzes the HMG-CoA biosynthetic reaction from acetoacetyl-CoA and HmgR catalyzes the mevalonate biosynthetic reaction from HMG-CoA. It has already been reported that overexpressing *hmgR* increased a carotenoid, lycopene, in a yeast *Candida utilis* mutant with an endogenous carotenoid synthetic pathway [11]. It was recently reported that overexpressing *hmgR* in *X. dendrorhous* increased astaxanthin production [10]. However, the effect of the other two genes involved in the mevalonate synthetic pathway, *acaT* and *hmgS*, on isoprenoid production was not evaluated.

**Figure 3:**
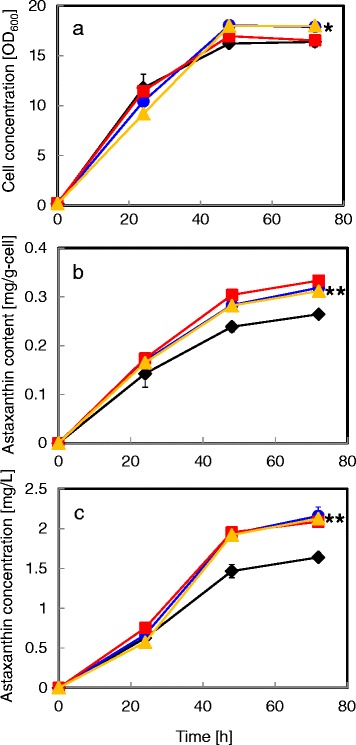
**Astaxanthin production by engineered strains overexpressing single mevalonate synthetic pathway genes. a** cell concentration (OD_600_); **b** intracellular astaxanthin content (mg/g-cell); **c** volumetric astaxanthin concentration (mg/L). *Diamond* (black), *Circle* (blue), *Square* (red) and *Triangle* (yellow) symbols represent values of the vector control strain, *acaT* overexpressing strain, *hmgS* overexpressing strain and *hmgR* overexpressing strain, respectively. The values are means and the error bars show the SD (n = 3). Student's *t*-test; asterisks indicate significant differences compared to the vector control strain (*P < 0.05, **P < 0.01).

In this study, we cloned and overexpressed *acaT* and *hmgS* to evaluate their effect on astaxanthin biosynthesis in *X. dendrorhous*. Previously, we showed the *gdh* promoter was an efficient promoter that showed higher expression level of target genes using GFP as a protein expression level indicator [7]. Therefore, we selected the *gdh* promoter to overexpress the *acaT*, *hmgS* and *hmgR* genes (Figure 2a). To compare the influence of single overexpression of *acaT*, *hmgS* and *hmgR* on astaxanthin production, three plasmids encoding each gene under the *gdh* promoter were constructed. *X. dendrorhous* mutant strains overexpressing *acaT*, *hmgS* or *hmgR* were obtained by transformation using these plasmids. We compared cell concentration, cellular astaxanthin content and volumetric astaxanthin concentration of these three mutant strains and the vector control strain (Figure 3). As shown in Figure 3a, the strains overexpressing *hmgS* and *hmgR* showed statistically higher final cell growth compared with the vector control strain. However, the *acaT* overexpressing strain showed almost equal cell growth to the control strain. The intracellular astaxanthin content of the strains overexpressing *acaT* (0.32 mg/g-cell) and *hmgS* (0.33 mg/g-cell) was 1.3-fold higher than that of the control strain (0.26 mg/g-cell) after 72 h of culture (Figure 3b). These values were equal to that of the strain overexpressing *hmgR* (0.31 mg/g-cell), which was previously shown to be critical to enhance the astaxanthin content [10,11]. With the increased intracellular astaxanthin content and normal cell growth in the strains overexpressing *acaT*, *hmgS* or *hmgR*, the volumetric astaxanthin concentrations (2.1-2.2 mg/L) after 72 h were equally increased compared with that of the control strain (1.6 mg/L) by about 1.3-fold (Figure 3c). This new insight proposes that the bottle-neck for intracellular mevalonate supply is not solely the reaction catalyzed by HmgR, but all three reactions involved in mevalonate biosynthesis reactions catalyzed by AcaT, HmgS and HmgR. This result is consistent with the previous result that indicated the presence of other rate-limiting enzymes from HmgR for isoprenoids biosynthesis in plant [12].

### Overexpression of multiple mevalonate synthetic pathway genes

Equal enhancement of astaxanthin production by each of the genes involved in the mevalonate synthetic pathway indicates the equal contribution of each reaction in this pathway to astaxanthin production in *X. dendrorhous*. Thus, to enhance astaxanthin production by improving mevalonate production, overexpression of multiple mevalonate synthetic pathway genes would be critical. To achieve this, plasmids including either G418 or Zeocin resistant genes and three cloning sites for target genes under strong promoters were constructed (Figure 2b). The strong promoters, P*act*, P*adh4* and P*tpi* were identified by our previous study [7]. Using this multiple gene expression system, construction of a strain overexpressing three mevalonate synthetic pathway genes (*acaT*, *hmgS* and *hmgR*) and a strain overexpressing only *hmgR* was carried out. Cell concentration, intracellular astaxanthin content and volumetric astaxanthin concentration were compared with the vector control strain after 72 h of culture (Table 1). All strains showed almost the same cell growth. The triple overexpressing strain, which overexpresses *acaT*/*hmgS*/*hmgR,* showed 1.4-fold higher volumetric astaxanthin production (2.5 mg/L) compared with that of the control strain (1.6 mg/l), although the *hmgR* single overexpressing strain still showed slightly higher astaxanthin production than the control strain (2.2 mg/L). As the result, triple overexpression of *acaT*, *hmgS* and *hmgR* using the multiple gene expression system developed in this study synergistically improved astaxanthin production without reducing cell growth.

**Table 1:** **Astaxanthin production by combined overexpression of mevalonate synthetic pathway genes in** ***X. dendrorhous***

**Overexpressing Gene(s)**	**Cell concentration [OD**_**600**_ **]**	**Astaxanthin content [mg/g-cell]**	**Astaxanthin concentration [mg/L]**
vector control	16.2 ± 0.34 (1.0)	0.26 ± 0.009 (1.0)	1.6 ± 0.09 (1.0)
*hmgR*	16.5 ± 0.04 (1.0)	0.36 ± 0.005 (1.4)	2.2 ± 0.03 (1.4)
*acaT*/*hmgS*/*hmgR*	17.0 ± 0.16 (1.0)	0.39 ± 0.006 (1.5)	2.5 ± 0.06 (1.6)

Samples were taken after culture for 72 h.

The culture conditions are described in “Methods”.

Parentheses represent the relative values.

The values are represented as mean ± SD (n ≥ 3).

### Combinatorial overexpression of genes involved in mevalonate, β-carotene and astaxanthin synthetic pathways

**Figure 4:**
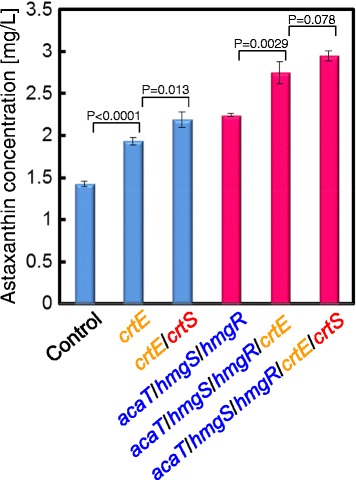
**Astaxanthin production by mutant strains overexpressing** ***crtE*** **and** ***crtE*** **/** ***crtS*** **.** Volumetric astaxanthin concentration (mg/L) of culture fermented for 72 h by the engineered strains overexpressing *crtE* gene or both *crtE* and *crtS* genes (*crtE*/*crtS*) with or without *acaT*/*hmgS*/*hmgR* overexpression. The values are means and the error bars show the SD (n = 3). The statistical difference between each gene-expression strain and its host strain analyzed by student’s *t*-test was shown as P-value.

CrtE catalyzes biosynthesis of geranylgeranyl-pyrophosphate (GGPP) from farnesyl-pyrophosphate (FPP) in the β-carotene synthetic pathway in *X. dendrorhous* (Figure 1). Breitenbach, *et al*., reported that self-cloning and overexpression of the *crtE* gene increased astaxanthin production [13]. To test the synergetic effect on astaxanthin production of enhancing synthetic pathways of mevalonate, β-carotene and astaxanthin, the *crtE* gene was overexpressed in either the parental strain or the *acaT*/*hmgS*/*hmgR* overexpressing strain using the multiple gene overexpression system. Comparing the volumetric concentration of astaxanthin produced by these strains, *crtE* overexpression increased the astaxanthin production in both the strains by about 1.3-fold (Figure 4). Additionally, combined overexpression of *crtE* and *crtS*, a gene in the astaxanthin synthetic pathway, synergistically increased astaxanthin production in both the parental strain and the *acaT*/*hmgS*/*hmgR* overexpressing strain by about 1.6-fold. The astaxanthin production of the *acaT*/*hmgS*/*hmgR*/*crtE*/*crtS* overexpressing strain after 72 h reached 3.0 mg/L, which was 2.1-fold higher compared with the control strain (1.4 mg/L). This result indicates that the increased mevalonate production platform strain overexpressing *acaT*/*hmgS*/*hmgR* has potential to enhance isoprenoids production when combined with additional overexpression of appropriate genes, depending on the target isoprenoid, using the multiple gene overexpression system developed in this study.

## Conclusion

In this study, we focused on developing a system to strongly express multiple genes in *X. dendrorhous* to improve isoprenoid production. We constructed two types of multiple gene expression vectors including either a G418 resistance gene or Zeocin resistance gene (Figure 2b). These vectors include three cloning sites under different strong promoters, P*adh4*, P*act* and P*tpi*, which were evaluated in our previous study [7]. Using this multiple gene overexpression system, we succeeded in synergistically improving astaxanthin production in *X. dendrorhous* through metabolic engineering to overexpress the three mevalonate synthetic pathway genes *acaT*, *hmgS* and *hmgR*, along with a β-carotene synthetic pathway gene (*crtE*) and an astaxanthin synthetic pathway gene (*crtS*). Astaxanthin is an end product of the isoprenoid synthetic pathway and its synthesis in this engineered strain indicates its ability to produce isoprenoids. Therefore, genetic engineering using the developed system to strongly express multiple target genes in a platform strain overproducing mevalonate has the potential to improve production of various value-added isoprenoids in *X. dendrorhous*.

## Methods

### Strains and media

NovaBlue (Novagen, Madison, WI, USA) was used as the *Escherichia coli* host strain for recombinant DNA manipulation. *Xanthophyllomyces dendrorhous* (NBRC 10129) was used as the parental host strain for gene expression. *E. coli* transformants were grown in LB medium (10 g/L tryptone, 5 g/L yeast extract, and 5 g/L sodium chloride) supplemented with 100 μg/mL ampicillin. Transformants of *X. dendrorhous* were cultured in YM medium (5 g/L tryptone, 3 g/L yeast extract, 3 g/L malt extract and 10 g/L glucose). Yeast extract and malt extract were purchased from Becton Dickinson (Sparks, MD, USA). Other chemicals were obtained from Nacalai Tesque (Kyoto, Japan) or Wako Chemicals (Osaka, Japan).

### Plasmid construction

Target genes were cloned by PCR using KOD-Plus-Neo DNA polymerase (Toyobo, Osaka, Japan). Nucleotide sequences of cloning primers for target genes are shown in Additional file 1. Construction of single or multiple gene expression vectors is shown in Figure 2. Nucleotide sequences of *acaT* and *hmgS* were detected by comparison to known sequences of *Cryptococcus neoformans*, whose amino acid sequence of *hmgR* has high homology with that of *X. dendrorhous* [5], using the BLASTX program through the Genome Net web site (http://www.genome.jp/tools/blast/). The sequences of *acaT* and *hmgS* have been registered in the GenBank with the accession numbers AB919149 and AB919150, respectively. Both these genes and the *hmgR* gene [10] were amplified by PCR from *X. dendrorhous* genomic DNA. The forward primer and reverse primer sets used for these amplifications were *acaT*-fw/*acaT*-rv, *hmgS*-fw/*hmgS*-rv and *hmgR*-fw/*hmgR*-rv, respectively. *Spe*I sites were introduced into the forward primers and *Stu*I sites were introduced into the reverse primers. The amplified fragments from *acaT*, *hmgS* and *hmgR* were digested with *Spe*I/*Stu*I and inserted into the *Spe*I/*Stu*I site of pKF-G418-G (Figure 2a) to construct pKF-G418-G-*acaT*, pKF-G418-G-*hmgS* and pKF-G418-G-*hmgR*, respectively.

The G-418 resistant multiple gene expression vector, pKF-G418-AAT (Figure 2b), was constructed by cloning the *act* promoter and terminator (P*act*/T*act*), P*adh4*/T*adh4* and P*tpi*/T*tpi* from *X. dendrorhous* genomic DNA by PCR using the In-Fusion® HD Cloning Kit (Takara, Shiga, Japan). The *acaT*, *hmgS* and *hmgR* genes were also amplified by PCR from *X. dendrorhous* genomic DNA using forward primer and reverse primer sets *acaT*-MCS3-fw/*acaT*-MCS3-rv, *hmgS*-MCS2-fw/*hmgS*-MCS2-rv and *hmgR*-MCS1-fw/*hmgR*-MCS1-rv, respectively. The *Asc*II/*Spe*I, *Sac*II/*Pac*I and *Mfe*I/*Mfe*I restriction sites were introduced into these forward/reverse primer sets. The amplified fragments from *hmgR* were digested with *Mfe*I and inserted into the *Mfe*I site in MCS1 of pKF-G418-AAT to construct pKF-G418-AAT-*hmgR*. The amplified fragment from *hmgS* was digested with *Sac*II/*Pac*I and inserted into the *Sac*II/*Pac*I site in MCS2 of pKF-G418-AAT-*hmgR* to construct pKF-G418-AAT-*hmgR*/*hmgS*. The amplified fragment from *acaT* was digested with *Asc*II/*Spe*I and inserted into the *Asc*II/*Spe*I site in MCS3 of pKF-G418-AAT-*hmgR*/*hmgS* to construct pKF-G418-AAT-*hmgR*/*hmgS*/*acaT*.

To construct the Zeocin resistant multiple gene expression vector pKF-Zeo-AAT, the Zeocin resistance gene was amplified by PCR from pREMI-z (Novagen) using the Zeo-BsiWI-fw/Zeo-BsiWI-rv forward and reverse primer set. The resulting Zeocin resistant gene fragment was cloned into the pKF-G418-AAT vector lacking the G418 resistance gene using the In-Fusion® HD Cloning Kit. A β-carotene synthetic pathway gene, *crtE,* and an astaxanthin synthetic pathway gene *crtS* were amplified by PCR from *X. dendrorhous* genomic DNA using forward primer and reverse primer sets *crtE*-MCS1-fw/*crtE*-MCS1-rv and *crtS*-MCS2-fw/*crtS*-MCS2-rv, respectively. *Mfe*I/*Mfe*I and *Sac*II/*Pac*I were introduced into these forward/reverse primer sets. The amplified fragments from *crtE* were digested with *Mfe*I and inserted into the *Mfe*I site in MCS1 of pKF-Zeo-AAT to construct pKF-Zeo-AAT-*crtE*. The amplified fragment from *crtS* was digested with *Sac*II/*Pac*I and inserted into the *Sac*II/*Pac*I site in MCS2 of pKF-Zeo-AAT-*crtE* to construct pKF-Zeo-AAT-*crtE*/*crtS*.

### Yeast transformation

All constructed plasmids were digested with *Nde*I at both the ends of rDNA regions (Figure 2) and transformed into the *X. dendrorhous* host strains to construct the target gene overexpressing strains. Transformation was carried out based on the method described in previous reports [14,15] with some modifications for construction of competent cells: *X. dendrorhous* parental strain was grown in 5 mL liquid YM medium at 22°C with agitation at 250 rpm for 24 h. An adequate volume of each culture was inoculated into 150 mL liquid YM medium to achieve an initial OD_600_ value of 0.03. Cultures were then grown at 22°C with agitation at 120 rpm for 16.5 h.

The *Nde*I digested plasmids, pKF-G418-AAT, pKF-G418-AAT-*acaT*, pKF-G418-AAT-*hmgR* and pKF-G418-AAT-*hmgS* were transformed into the parental *X. dendrorhous* host strain to construct the vector control strain, *acaT* overexpressing strain, *hmgS* overexpressing strain and *hmgR* overexpressing strain, respectively, and compare their cell growth and astaxanthin production (Figure 3). The *Nde*I digested plasmids, pKF-G418-AAT, pKF-G418-AAT-*hmgR* and pKF-G418-AAT-*acaT*/*hmgR*/*hmgS* were transformed into the parental *X. dendrorhous* host strain to construct the vector control strain, *hmgR* single overexpressing strain and *acaT*/*hmgR*/*hmgS* multiple overexpressing strain, respectively, and compare their cell growth and astaxanthin production (Table 1). The *Nde*I digested plasmids, pKF-Zeo-AAT, pKF-Zeo-AAT-*crtE* and pKF-Zeo-AAT-*crtE*/*crtS* were transformed into the pKF-G418-AAT vector control strain or the *acaT*/*hmgR*/*hmgS* multiple overexpressing strain to construct the vector control strain, *crtE* overexpressing strain, *crtE*/*crtS* overexpressing strain, *acaT*/*hmgR*/*hmgS* overexpressing strain, *acaT*/*hmgR*/*hmgS*/*crtE* overexpressing strain and *acaT*/*hmgR*/*hmgS*/*crtE*/*crtS* overexpressing strain and compare their cell growth and astaxanthin production (Figure 4).

### Cultivation of *X. dendrorhous* strains

*X. dendrorhous* strains overexpressing target genes were grown in 5 mL liquid YM medium containing 40 μg/mL G418 and 200 μg/mL Zeocin if needed in test tubes at 22°C with agitation at 250 rpm for 72 h. An adequate volume of each culture was inoculated into 80 mL liquid YM medium in a Sakaguchi Flask to achieve an initial OD_600_ value of 0.15. Cells were then grown at 22°C with agitation at 120 rpm for less than 72 h.

### Analytical methods

Cell concentration was measured as the optical density at 600 nm after culture for the appropriate time. To measure the intracellular astaxanthin content of *X. dendrorhous* mutants, harvested cells were suspended into 1 mL acetone. The cells were broken using a bead shocker (Shake Master NEO, bms, Tokyo, Japan) with zirconia beads. The cell extract was centrifuged at 15,000 × *g* at 4°C for 10 min, and then the supernatant was diluted into an appropriate volume of acetone. Astaxanthin concentration was determined using a high performance liquid chromatography (Shimadzu, Kyoto, Japan) equipped with a Develosil ODS-HG-5 column (Nomura Chemical, Aichi, Japan). The operating conditions were 25°C, with acetonitrile/methanol/2-propanol (85/10/5 (v/v)) as the mobile phase at a flow rate of 0.8 mL/min, and the detection was performed at 471 nm with a UV detector SPD-20A (Shimadzu).

## Additional file

Additional file 1:
**Nucleotide sequence of cloning primers in this study.**
